# Advances in research concerning the association between allergic rhinitis and chronic sinusitis and cognitive impairment

**DOI:** 10.3389/fneur.2026.1759585

**Published:** 2026-05-04

**Authors:** Yu Chen, Guohong Jiang, Xiaohe Zhang, Zhiwei Zhou, Ping Xu

**Affiliations:** 1 Affiliated Hospital of Zunyi Medical University, Zunyi, China; 2 The Second Affiliated Hospital of Zunyi Medical University, Zunyi, China

**Keywords:** allergic rhinitis, chronic sinusitis, cognitive fuction, cognitive impairment, sleep disorder

## Abstract

As the prevalence of chronic rhinosinusitis (CRS) and allergic rhinitis (AR) continues to rise worldwide, more and more patients are reporting a lower quality of life. In addition to causing common nasal symptoms such as congestion and rhinorrhea, these illnesses also show a close association with cognitive function in several ways. However, the available evidence remains dispersed. This paper reviews existing research on the effects of AR and CRS on cognitive function, organized into five sections: disease characteristics, cognitive impacts, mechanism pathways, therapeutic interventions, and future directions. Clinical research suggests that AR has a significant association with children’s academic performance, neurodevelopment, and cognitive function, with a potential correlation with cognitive impairments. Additionally, adults’ anxiety, depression, and cognitive impairments show a negative correlation with AR and CRS. Numerous factors, such as sleep disturbances, the intensity of facial symptoms, the nose-brain communication axis, brain activity and connectivity, and other issues, may be linked to the causes of cognitive impairment. Furthermore, there has been some advancement in the study of how AR and CRS treatments, such as sinus surgery, intranasal corticosteroids, and antihistamines, affect cognitive function. This data suggests that AR and CRS are closely related to cognitive impairment. However, there is still a lack of solid research evidence connecting AR and CRS to cognitive dysfunction, and further exploration is needed regarding their pathogenesis and pharmacological treatment.

## 1 Overview

### 1.1 Definition and epidemiology

Allergic rhinitis (AR) refers to a condition in which the nasal mucosa, upon exposure to allergens, triggers IgE-mediated Type 2 inflammation, leading to nasal symptoms. Rhinorrhea, nasal congestion, nasal itching, and sneezing are among its common clinical manifestations. With up to 33% of AR patients experiencing sleep disorders, there is a markedly increased risk of anxiety, depression, and suicidal thoughts and behaviors (1). According to research, children frequently suffer from AR. According to a 2022 report, 22% of Chinese children have AR (2), while the global prevalence of AR increased from 8.39% between 2012 and 2015 to 19.87% between 2016 and 2022. Among adults, the global prevalence of AR is approximately 18.1%, ranging from 3.6 to 22.8% in Africa, 1.0 to 43.9% in Europe, 19.2 to 47.5% in Oceania, 1.0 to 47.9% in Asia, and 3.5 to 54.5% in the Americas (2). The overall prevalence of AR has increased over the past several decades (3, 4). Multiple air pollutants show significant associations with AR, with varying strengths of association across different pollutants, regions, and levels of development. Children in China who are exposed to NO₂ have a higher risk of developing AR (5, 6).

Chronic rhinosinusitis (CRS) refers to inflammation of the sinus mucosa lasting 12 weeks or longer. Its pathogenesis involves chronic impairment of epithelial barrier function due to genetic predisposition, immune deficiencies, and environmental factors; activation of T and B cells; persistent inflammatory responses (7) and tissue remodeling; and biofilm formation (8–11). Its clinical manifestations include nasal congestion, craniofacial pain, olfactory dysfunction, sleep disturbances, and emotional disorders, significantly impairing patients’ quality of life (12). Although both AR and CRS involve inflammatory immune responses, studies indicate that CRS can manifest three primary inflammatory subtypes. Furthermore, the mechanisms of B-cell activation differ between AR and CRS. In AR, IgE is predominantly oligoclonal and antigen-specific, whereas in CRS, it is primarily polyclonal and non-antigen-specific (13). These distinctions may influence the mechanisms underlying cognitive impairment, as well as variations in treatment approaches and outcomes. CRS is classified into two types based on the presence of nasal polyps: CRS with nasal polyps (CRSwNP) and CRS without nasal polyps (CRSsNP). About one-third of patients have the polyp-associated type, and most of them report nasal congestion as their most bothersome symptom (14). It can also be classified as diffuse or localized, primary or secondary (15), and more researchers are starting to categorize it according to pathophysiological subtypes. According to a recent report, the prevalence of CRS has risen globally from 4.72% in 1980–2000 to 19.40% in 2014–2020 (16). In the United States, healthcare costs have increased to approximately $144 billion annually (17). Due to factors such as genetics, demographics, and environment, as well as variations in diagnostic criteria, the prevalence and subtypes of CRS differ across the globe. Consequently, the racial, gender, and age distributions of patients also vary significantly depending on the region and subtype (15, 18, 19).

In conclusion, patients’ quality of life is greatly impacted by AR and CRS, which have emerged as a global health concern. These conditions are increasing the global economic burden as their prevalence rises.

### 1.2 AR and CRS: association with cognitive impairment

In addition to nasal-specific symptoms, AR and chronic sinusitis also cause extra-nasal symptoms, such as cognitive impairment, which have a substantial adverse impact on patients’ health and productivity at work. The prevalence of AR among adults is approximately 7.9%, while CRS affects about 4.9% of the population. Beyond cognitive impairments, AR patients experience greater limitations in work and social activities compared to non-AR individuals, whereas CRS patients face more restrictions in physical activities and employment (20). As early as 1999, scholars noted that AR can lead to complications including cognitive impairment, impaired hearing and language development, and sleep-disordered breathing (21). Additionally, studies have shown that ragweed season has an impact on the working memory of AR patients (22). Current research also indicates that patients with chronic sinusitis may exhibit a potential underlying basis for reduced functional brain connectivity associated with cognitive function (23). CRS shows a close association with a decline in patients’ cognitive function, with the severity of the deterioration closely related to the quality of life and symptom severity. Interestingly, asthma shares the same category of airway allergic diseases as AR and CRS. Current research also indicates that childhood asthma correlates with memory impairments. Early-onset asthma may slow the development of episodic memory, leading to greater deficits in processing speed and attention (24, 25). Among the elderly population, asthma patients are more prone to developing mild cognitive impairment (26).

Furthermore, current survey data indicate that AR has adverse effects on socioeconomic outcomes, work efficiency, sleep patterns, and learning difficulties (27). AR also impacts patients’ cognitive functions, academic performance, behavior, and quality of life (28). Additional research suggests associations between AR and Attention Deficit Hyperactivity Disorder (ADHD) as well as autism spectrum disorders (29, 30). AR has a substantial negative influence on patients’ mental health in addition to their quality of life (31). According to a wealth of research, AR is linked to anxiety and depression, and these outcomes may be exacerbated by immune system impairment, sleep disorders, and cognitive dysfunction (32). Additionally, research indicates that CRS patients are more likely to experience depression, and their caregivers may experience an even higher psychological burden (33).

Therapeutically, first-generation (sedating) antihistamines’ central nervous system (CNS) side effects negatively impact cognitive functions such as memory and reaction time. In contrast, second-generation (non-sedating) antihistamines and intranasal corticosteroids may improve cognitive function (detailed comparison in Section 4.1). For patients with refractory or complicated CRS, endoscopic sinus surgery, in addition to corticosteroids and anti-inflammatory medications, can also contribute to cognitive improvement.

Regarding the impact of AR and CRS disorders and their therapies on cognitive function, recent clinical research has made some strides. There are still a few divergent opinions regarding their effect on cognitive function, though. Further investigation is required to clarify the underlying mechanisms, as basic experimental studies in this field have only produced limited results.

## 2 The impact of AR and CRS on cognitive function

### 2.1 The association between AR and children’s cognitive functions and neurodevelopment

Current research indicates that AR exerts a certain correlation with children’s cognitive functions, including memory and reaction speed. Children with allergic airway diseases, such as AR, had higher memory problems, decreased play activities, and a higher percentage of special education needs, according to a 2014–2017 survey on children’s health (34). According to a separate Swedish study, after effectively controlling for medication and seasonal confounders through standardized drug regimens, strict seasonal window delineation, pollen exposure monitoring, and matched controls, children with AR had significantly performed worse on memory, reaction speed, and motor attention tests during pollen season compared to healthy controls. The study also identified a correlation between simple reaction time and symptom scores (35).

Regarding neurodevelopment, a survey involving 109,842 samples of children with atopic dermatitis (AD) who experienced AR revealed a significant association between childhood AD and self-reported memory impairment risk. Developmental delays were also observed in children with AR (36). These results are consistent with another Korean study that included 30,557 children with AD and 89,452 controls. The study found that children with AD had increased risks of neurodevelopmental disorders in both fine and gross motor skills. The likelihood of neurodevelopmental disorders in the language domain was higher in male children. The study also identified that AD elevated risks of intellectual disability and behavioral disorders (37). When analyzing the association between AD and neurodevelopmental dysfunction, the study adjusted for systemic steroid use and antihistamine exposure to account for potential confounding effects of medication. This data suggests that, in addition to its effects on cognitive function, AR may be a substantial risk factor for neurodevelopmental delays in children.

Regarding academic performance, Marcotte et al. analyzed data from 16 allergy reporting stations and 48 school districts across the United States, focusing on students in grades three through eight. Their research on the relationship between seasonal AR and academic achievement revealed that higher regional pollen levels correlated with lower-than-expected scores in mathematics and English language arts. This phenomenon was especially noticeable in the math assessments of lower-grade students (38). In this study, the effects of seasonal confounding were minimized through a fixed-effects and time-varying design. Using a student fixed-effects model and conducted a placebo test to indirectly account for confounding effects of medication and seasonal factors, Bensnes found in another study that pollen exposure had a negative impact on the test scores of high school students. Students’ test scores decreased by 2.5% for every standard deviation increase in pollen levels (39). Although numerous studies indicate that academic performance is affected by rhinitis, the presence of numerous confounding factors influencing academic outcomes introduces potential bias. Therefore, these findings require further validation through standardized research designs.

Only a few studies have produced contradictory results. For example, a Taiwanese cross-sectional study involving 109 children with AD who also had AR (87.2% of the AD cohort) and a healthy control group found no differences in cognitive scores. Additionally, the study found no connection between ADHD and allergic diseases (33). The small sample size could be one of the causes of these results.

### 2.2 The association between AR and adult cognitive function

Numerous clinical studies on AR and CRS in adult patients have also been carried out, in addition to advancements in pediatric research. A cross-sectional study involving 100 adult patients with AR and 96 controls found that, after excluding participants who had used systemic steroids within the past month, the ratio of attention difficulties in the AR group versus the control group was 65 to 34.4% (40). In a similar vein, Wilken et al. (41) found that, compared to the control group, participants exhibited increased drowsiness and decreased reaction speed, alertness, and mental clarity following ragweed pollen-induced AR symptoms. In this study, the effects of medication and seasonal variables on the results were controlled through standardized drug administration, randomized grouping, baseline symptom screening, and conducting the research during the non-allergy season. Although these studies all indicate that adult AR patients exhibit declines in cognitive performance, such as attention and reaction time, the correlation between AR symptom severity and its association with cognitive function remains controversial. Subjects with AR symptoms exhibited a lower quality of life, higher rates of work absenteeism, more frequent sleep disturbances, and lower cognitive test scores in another questionnaire survey involving 7,024 participants. However, unlike previous findings, cognitive scores deteriorated progressively with increasing severity of AR symptoms (42).

AR patients often struggle with communication, in addition to experiencing difficulty paying attention and reacting quickly. A clinical study from Turkey on the communication abilities of students with AR demonstrated that, after controlling for medication and seasonal allergen effects on assessment, AR patients scored 29.58 ± 5.94 on the Social Communication Skills Rating Scale, and 17.16 ± 2.88 on the Communication Questionnaire, compared to 36.22 ± 7.00 and 13.16 ± 3.04 in the control group, respectively (43). In addition, allergies may increase traffic fatalities by impairing cognitive function. A study by Danagoulian et al. demonstrated that pollen allergy can significantly raise the risk of traffic fatalities by inducing cognitive deficits, including prolonged reaction time and reduced attention. Furthermore, alcohol use exhibits a synergistic effect that further amplifies this risk (44). Current studies are underway to develop treatment approaches that enhance safety in automotive and aviation operations.

Current research has shown that AR patients can make compensatory adjustments to better complete tasks, in addition to the detrimental effects on cognitive-related functions. A study from the Netherlands indicates that AR patients perform worse in mental health assessments, sustained attention tests, and reaction tests. Interestingly, the research also found that AR patients showed no difference from the control group in short-term cognitive performance tests, yet required significantly greater mental effort (45). This implies that AR patients may use compensatory effort to compensate for cognitive decline in short-term tasks. Regarding compensatory adjustments in AR patients, Trikojat et al. (46) found that while AR patients exhibited reduced sustained attention, reaction time, and other cognitive abilities, they also demonstrated greater adaptability in controlling their attention. Trikojat et al. found that AR patients performed worse on immediate and delayed memory tasks and had significantly lower word recall when compared to non-allergic controls. However, the research also revealed that AR patients modified their testing methods during dual-task testing (47). This may be the result of AR patients improving their attentional regulation to compensate for attention deficits. It is noteworthy that these studies effectively controlled for confounding effects of medication and seasonal factors on cognitive function assessments through rigorous measures in their experimental designs. These included strict exclusion of interfering medications, controlled drug exposure, testing conducted during non-allergic seasons, standardized nasal provocation tests, seasonally stratified testing, and matched sample groups.

Similar to pediatric studies, some opposing views have emerged in adult patient research, suggesting that the effects of AR on cognitive function remain controversial and require further investigation to gather additional data. A Swiss study, after meticulously controlling for medication classification, seasonal stratification, and multidimensional covariate adjustments to effectively mitigate confounding factors related to drug therapy and seasonality, assessed participants’ cognitive daily performance during the pollen season over 10 consecutive days. The findings revealed that only under high pollen exposure did participants exhibit slightly slower reaction times in the grammatical reasoning test, while no significant differences were observed in scores across the four cognitive assessment tests (48). In addition, Hartog et al. (49) observed no appreciable variations in cognitive speed or memory retrieval between patients with severe AR and healthy controls. Similarly, a twin case–control study examining atopic conditions and dementia found that a history of atopic conditions was associated with an increased risk of Alzheimer’s disease. Still, no significant differential findings were observed in the separate analysis of rhinitis (50). According to other research, AR patients’ cognitive abilities, including memory, retrieval, and information processing, do not change, even though they have greater rates of anxiety, depression, and sleep disorders (31, 51). The reasons for these contradictory findings may stem from multiple factors, including the low reliability of self-reported rhinitis, racial differences among study subjects, and variations in survey questionnaires and cognitive function assessment criteria.

### 2.3 The effects of CRS on adult cognitive function

Research on the effects of CRS on children’s cognitive functions remains relatively scarce, and the direct association between CRS and children’s attention, learning abilities, and neurodevelopment has yet to be clearly established. In adult studies, CRS patients performed worse on the Cognitive Failure Questionnaire (CFQ), a self-reported tool that assesses an individual’s subjective perception of cognitive failures and their impact on daily functioning, and the Simple Reaction Time Test, according to a prospective case–control study by Soler et al. (52) with CRSsNP showing more pronounced effects than CRSwNP. In this study, the authors excluded CRS patients who had used oral corticosteroids within the past two weeks or undergone sinus surgery within the past six months by controlling for antihistamine use, thereby minimizing the interference of medication and surgery on cognitive function. Studies by Cvancara et al. (53) also indicate that CRS patients exhibit a higher incidence of cognitive impairment, with lower Montreal Cognitive Assessment (MoCA) scores and reduced accuracy in eye-tracking tests.

However, similar to AR, a small number of scholars have raised questions about this. For example, in a cross-sectional study by Neil, subjective cognitive test results reported by patients showed that while AR patients had a higher risk of cognitive limitations, there was no significant association between CRS patients and cognitive functional impairments. This may be attributed to the distinct pathological mechanisms underlying the effects of AR and CRS on cognitive function, as well as the absence of quantitative scoring in the cognitive tests employed in this study (20).

### 2.4 Cognitive impairment caused by AR and CRS is associated with accompanying symptoms

A relevant clinical study also found a correlation between patients’ sinus-related quality of life and cognitive test results (53). Similarly, in a cross-sectional survey by Tarasidis et al. (54) involving 70 CRS patients, after controlling for pre-enrollment treatments to minimize the short-term impact of medications on cognitive function and pain assessments, correlations were demonstrated between disease-specific quality of life measures and scores on the CFQ in CRS patients. Current research also indicates that the olfactory specificity parameters of CRS, olfactory dysfunction, and MoCA scores are correlated with the prevalence of MCI. Cognitive impairment may be somewhat predicted by olfactory discrimination scores (55). Facial pain associated with CRS, as demonstrated in the study by Cvancara et al. (54) showed positive correlations between the scores of CRS patients on the Brief Pain Inventory-Short Form, the Brief McGill Pain Questionnaire, and the CFQ. These results show that patients with CRS not only experience cognitive decline, but also that the degree of cognitive impairment is correlated with the severity of symptoms and the impact on quality of life.

In addition to quality of life, recent studies have demonstrated that AR and CRS have distinct effects on the mental health of adult patients. In a cross-sectional survey, Fereidouni et al. (51) discovered that adult female AR patients scored higher on the Depression, Anxiety, and Stress Scale (DASS-21), quality of life surveys, and insomnia questionnaires compared to non-allergic individuals. According to a case–control study by Rodney et al. (56) non-polypoid CRS had a more noticeable effect than polypoid CRS, and the prevalence of depression among CRS patients was roughly twice that of the control group. Through their impact on sleep disorders, endocrine disruptions, and other associated factors, these psychological abnormalities may have an indirect effect on cognitive function.

## 3 Mechanisms of cognitive impairment caused by AR and CRS

### 3.1 Common mechanism

**Figure 1:**
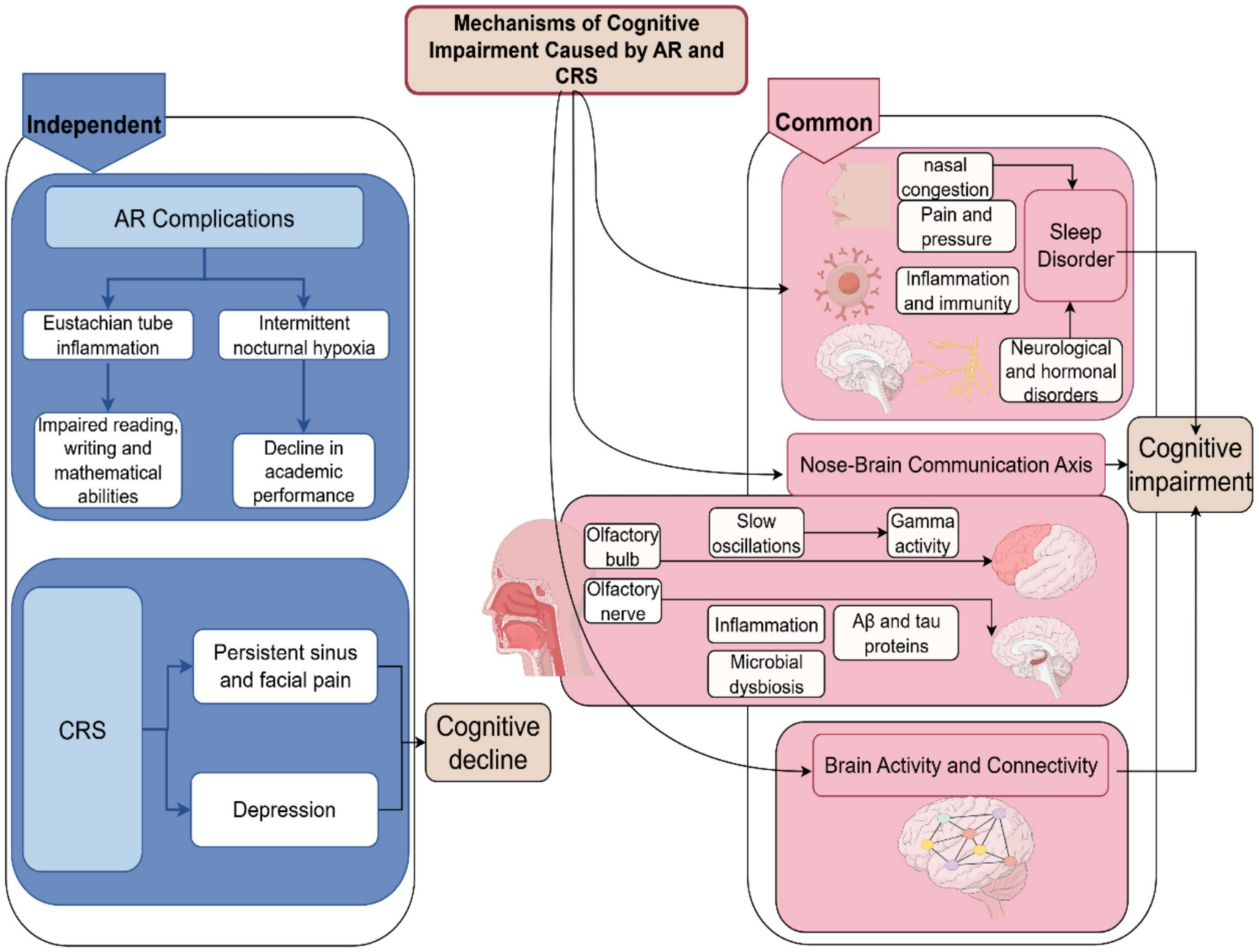
Mechanisms of cognitive impairment caused by AR and CRS (by Figdraw).

Mechanisms of cognitive impairment caused by AR and CRS as shown in Figure 1.

#### 3.1.1 Sleep disorders

According to recent studies, obstructive sleep apnea syndrome and other sleep disorders can cause cognitive decline. Patients with AR and CRS face a significant risk of severe sleep disturbances, which are major contributors to daytime sleepiness and fatigue. These issues are closely linked to quality of life and mental health (57). Sleep disturbances caused by AR and CRS may represent a direct mechanism of cognitive impairment. Research shows that during allergy seasons, AR patients have more micro-awakenings. CRS patients have abnormal subjective sleep parameters, such as the Pittsburgh Sleep Quality Index (PSQI) and Fatigue Severity Scale (FSS), as well as abnormal objective sleep parameters, such as minimum oxygen saturation, apnea-hypopnea index (AHI), and respiratory disturbance index (RDI), in comparison to normal values, according to a statistical analysis of people with abnormal sinus-specific questionnaire scores (58).

The mechanisms by which they cause sleep disturbances include: (1) Symptoms such as coughing, sinus pain/pressure, and headaches induced by the disease; (2) Nasal obstruction: Nasal mucosal congestion narrows the nasal passages, increasing total upper airway resistance. The horizontal position during sleep further elevates resistance, leading to turbulent airflow, pharyngeal collapse, and snoring, which in turn trigger micro-awakenings (59); (3) Inflammation and immune dysregulation: Upregulation of “anti-sleep” Th2-associated inflammatory cytokines like interleukins (IL), IL-4, IL-10, transforming growth factor-*β*, and IL-13, along with abnormal histamine release; (4) Dysfunction of sympathetic/parasympathetic nervous system; (5) Additionally, circadian rhythm hormones like melatonin play a role in the relationship between AR and sleep disorders. Melatonin is a key neurohormone regulating circadian rhythms. Exogenous melatonin not only improves sleep but also alleviates AR symptoms by suppressing immune responses, reducing IgE levels, and attenuating nasal mucosal inflammation (60). Asthma shares similarities with AR and CRS in this regard. Some scholars suggest that asthma may also increase neurocognitive risks through neuroinflammation, hypoxia, and other mechanisms, particularly affecting memory functions associated with the hippocampus (61).

#### 3.1.2 Nose-brain communication axis

Through neuroimmune mechanisms, the nose-brain communication axis can control both the immune system and the central and peripheral nervous systems. Current research suggests that its impact on cognition may be associated with reduced oscillation frequencies in brain regions, disrupted functional coupling between the ventral hippocampus and anterior cingulate cortex, the release of inflammatory cytokines, and the expansion of Aβ and tau proteins (62). Studies reveal both structural and functional abnormalities in the olfactory bulb of AR patients. The olfactory bulb controls slow oscillations that are synchronized with breathing throughout the brain, including the hippocampus and prefrontal cortex, during nasal breathing. Gamma activity linked to cognitive processes is modulated by these oscillations. Thus, the authors postulate that in AR patients, nasal blockage or damage to the olfactory bulb weakens synchronized respiratory oscillations in brain regions related to cognition, resulting in cognitive impairment (63). In basic research, Salimi et al. (64) found that ovalbumin-induced AR rats may exhibit enhanced anxiety-like behavior through functional coupling between the olfactory bulb and medial prefrontal cortex. Similarly, Rodrigues et al. noted that olfactory sensory neuron axons terminate in the olfactory bulb, and olfactory bulb pyramidal cells project to the olfactory cortex. The olfactory cortex maintains critical connections with the prefrontal cortex, a cognitive hub. Following ovalbumin-induced AR in rats, alterations were observed in the synaptic structure and activity of the olfactory bulb, anterior olfactory nucleus, and prefrontal cortex, with male rats exhibiting reduced aggression. However, no alterations in learning, memory, or anxiety levels were observed in these AR rats, which the authors attributed to the duration of AR induction (65). In relation to the inflammatory cascade response, Soltani et al. found that AR mice exhibited elevated expression of M1-polarized microglia in hippocampal tissue, decreased memory and learning capacities, and increased depressive and anxiety-like behaviors. Activation of the TLR4/NF-κβ inflammatory pathway may be linked to these abnormalities (66). In studies of CRS patients, another review suggests that Aβ and tau proteins can propagate through synapses. Aβ deposits may travel from nasal olfactory neurons to the CNS, possibly contributing to Alzheimer’s Disease, as the nasal cavity apex is close to olfactory brain regions. Furthermore, with aging, damage to the nasal epithelium, along with inflammation and microbial dysbiosis within the nasal cavity, may migrate to the CNS, contributing to the onset of neurodegenerative diseases (67).

#### 3.1.3 Brain activity and connectivity

AR patients exhibited lower amplitude, low-frequency oscillations in the precuneus (PCUN) and higher amplitude, low-frequency oscillations in the anterior cingulate cortex (ACC), according to a study by Gao et al. using resting-state functional magnetic resonance imaging to assess brain function in AR patients. Additionally, this disparity was linked to particular IgE levels, the visual analog scale, and the Rhinoconjunctivitis Quality of Life Questionnaire (RQLQ) (68). Due to altered brain function in these cognitively relevant regions, AR patients may experience memory impairment, attention deficits, and anxiety/depression. This is because PCUN and ACC activity are closely linked to cognitive function, memory, and emotional disorders. Additionally, cognitive, executive, and memory tasks involve the frontoparietal network (FPN), a component of the brain’s functional connectivity. Reduced FPN connectivity was observed in patients with sinusitis in a case–control study examining functional connectivity; the degree of sinusitis inflammation was correlated with this difference. According to the study, changes in frontoparietal network connectivity may indicate subclinical, early-stage changes in brain function because they are more sensitive than behavioral responses (23). Similar to these findings, abnormal dynamic characteristics of brain functional connectivity are also observed in asthma patients (69).

### 3.2 AR and CRS: independent mechanisms leading to cognitive impairment

#### 3.2.1 AR complications leading to cognitive impairment

In addition to the previously mentioned mechanisms, some researchers have proposed that children’s academic performance may be impacted by complications related to AR. For example, children’s reading, writing, and math skills may be affected by secondary eustachian tube inflammation. Children’s declining academic achievement has also been associated with intermittent nocturnal hypoxia brought on by AR (70).

#### 3.2.2 CRS chronic pain-induced cognitive impairment

One of the main clinical signs of CRS is persistent sinus and facial pain. A 2015 study examining the correlation between pain and cognitive function in adult CRS patients found associations between the CFQ and pain questionnaires such as the Brief Pain Inventory-Short Form (BPI-SF). One possible explanation for this association is chronic inflammation (54). However, there is still a dearth of research on the cognitive impairment brought on by facial pain in people with AR, and the underlying mechanisms are still unknown, necessitating more study. Additionally, research indicates that depression in patients caused by CRS may also play a role (52, 71). Unfortunately, while these findings demonstrate correlations with cognitive function, no further studies have been conducted on the specific mechanisms involved.

## 4 The impact of rhinitis treatment on cognitive impairment

### 4.1 The impact of AR therapy on cognitive impairment

Because different medications have distinct mechanisms of action, they exhibit varying effects on cognitive function when used to treat rhinitis. First-generation antihistamines, for instance, readily cross the blood–brain barrier (BBB) due to their high lipophilicity. They may worsen mood swings, cognitive decline, and drowsiness because of their sedative effects and other adverse effects on the CNS. Second-generation antihistamines, on the other hand, have trouble crossing the BBB because of their lipophobic properties and large side chains (72), and they may have negligible or no neuro-related adverse effects. When compared to second-generation antihistamines, diphenhydramine causes patients to be less alert, with more noticeable declines in delayed memory, more complaints of drowsiness, and higher levels of fatigue. It impairs working memory and attentiveness (72, 73). Another study also found that the second-generation medication desloratadine had less adverse effects on alertness and cognitive function and led to less drowsiness in AR patients compared to the first-generation antihistamine diphenhydramine (74).

Since they are currently the first-line treatment for patients with AR, second-generation antihistamines have garnered a lot of interest and study. According to a survey by Gandon et al., levocetirizine, which has a higher affinity for H1 receptors, did not significantly alter the critical flicker threshold or selective reaction time when compared to the placebo group. Furthermore, it showed increased alertness (73). Similarly, Valk et al. (75) found that, when compared to a placebo, the second-generation antihistamine bilastine 20 mg, administered in hypoxic conditions at 2438.4 m, did not affect alertness, drowsiness, or the ability to perform complex tasks. Even though second-generation antihistamines have less sedative effects, some medications still have some sedative qualities (76), and it’s still unclear how they affect the CNS in different situations. For instance, a Hungarian study found that at both ground level and 4,000 meters above sea level, 20 mg of bilastine did not significantly alter cognitive function when compared to a placebo group. However, at 4000 meters above sea level, the second-generation medication cetirizine reduced performance on divided attention tests, leading to more mistakes (77). Consistent with this finding, at 3048.0 m and 4572.0 m, cetirizine produced lower attention levels and slower reaction times. In contrast, nelfinavir showed no significant differences compared to placebo in reaction times or attention effects across various barometric pressure conditions (78). These studies indicate that hypoxic environments at high altitudes reduce blood oxygen saturation in individuals. Although hypoxia alone does not directly cause cognitive impairment, it may exacerbate cognitive deficits when combined with certain second-generation antihistamines. This suggests that hypoxia may amplify the central inhibitory effects of some medications, although the underlying mechanism remains to be further elucidated. Furthermore, when taken in varying dosages, the new generation of medications may also impair the CNS. Higher doses of loratadine have been shown in studies to impair significantly and have sedative effects on tests like choice reaction time when compared to a placebo (79, 80). These results might suggest that the CNS side effects of second-generation antihistamines, including sedation, slowed reactions, and cognitive impairment, are more easily revealed under hypoxic or high-dose conditions. Cetirizine appears to have this effect most prominently under hypoxic conditions. Therefore, clinicians must comprehensively consider the potential adverse effects (e.g., sedation, insomnia, restlessness, impaired attention, even mental disorders) for children, adolescents, and workers in high-concentration professions, as well as external factors that may exacerbate such effects, when formulating treatment plans.

Other drugs that do not cross the BBB may exert distinct effects on cognitive function. For instance, leukotriene receptor antagonists, which exhibit extremely low BBB penetration, have not been identified in clinical studies to directly induce cognitive abnormalities or CNS side effects. Instead, they can improve sleep quality in patients with AR by alleviating allergic inflammation and relieving nasal congestion (59), thereby ameliorating cognitive impairment indirectly. In addition, corticosteroids may exert distinct effects on cognitive function depending on the route of administration. For example, frequent or chronic use of oral corticosteroids can cross the BBB through the systemic circulation, exerting adverse effects on brain structure, function and cognitive performance, and may also induce psychological alterations such as anxiety and depression in adolescents. In contrast, inhaled and intranasal corticosteroids act primarily locally on the nasal mucosa with extremely low systemic bioavailability, and thus barely cross the BBB. Nevertheless, they can alleviate nasal congestion and improve sleep quality, which may consequently exert an indirect ameliorative effect on cognitive function (70). In a study by Mansfield et al. (81) it was also found that among AR patients, those in the intranasal fluticasone propionate treatment group exhibited improvements in nasal congestion and drowsiness compared with the placebo group, with shortened reaction times in cognitive tests, which ameliorated alertness to a certain extent. Notably, the duration of drug administration may also exert a certain impact on cognitive function. Studies have shown that the longer the duration of inhaled corticosteroid use, the higher the risk of cognitive impairment in patients with bronchial asthma (82).

### 4.2 The effects of CRS treatment on cognitive impairment

#### 4.2.1 The effect of pharmacotherapy on cognitive impairment caused by CRS

There are fewer studies on how medication affects cognitive function in CRS patients than there are for AR patients receiving drug treatments. The main medication regimens consist of hormones and anti-inflammatory medications. In a prospective study, 27 adult patients with CRS showed improvements in fatigue and sleep quality after receiving oral antibiotics, topical and systemic steroids, and sinus irrigation for six weeks. Subjective cognitive impairment questionnaire scores dropped significantly from 38.6 ± 16.4 to 33.0 ± 14.4. Improved executive function and reaction times were also shown by objective cognitive testing (83). Antihistamines, as a component of pharmacotherapy for CRS, may also have a certain correlation with cognitive function in CRS patients. A study by Soler et al. found that antihistamines exert a notable effect on subjective cognitive function assessed by the CFQ in CRS patients: the CFQ scores of CRS patients were significantly higher than those of the control group without antihistamine intervention, while after antihistamine administration, the overall CFQ score difference between the CRS group and the control group was no longer statistically significant (52).

In addition to hormones, anti-inflammatory drugs, and antihistamines, the impact of treatment options including vasoconstrictors, mast cell stabilizers, and immunotherapy on cognitive function still requires further research.

#### 4.2.2 The impact of endoscopic sinus surgery on cognitive impairment in CRS

For patients with CRS who are refractory to medication or have severe complications, endoscopic sinus surgery may be considered as an additional treatment option. This procedure can significantly improve CRS symptoms and associated problems like sleep disturbances by eliminating diseased tissue, decreasing nasal obstruction, and reducing inflammation and facial pain (57). Cognitive function is also positively impacted by endoscopic sinus surgery. In a study by Yoo et al., 33 patients with drug-resistant CRS exhibited significant improvements following surgical intervention, as evidenced by a marked decrease in 22-Item Sino-Nasal Outcome Test (SNOT-22) scores (from 54.8 ± 21.4 to 24.8 ± 21.1). Mathematical processing and sample matching tests also showed substantial improvement. CFQ scores (from 46.7 ± 18.4 to 31.9 ± 17.8) even reached levels comparable to healthy controls. The study also showed that the improvement in the non-polypoid group was more noticeable than in the polypoid group (84). Patients with CRS who underwent surgical treatment demonstrated improvements in their overall CFQ scores, SNOT-22 scores, and Health Questionnaire test results, according to another clinical study involving 247 adult patients. The improvement in the polyp-free group’s overall CFQ score, however, was not statistically significant, as reported in this study (71).

## 5 Summary and outlook

In conclusion, as the prevalence of AR and CRS increases, they progressively diminish patients’ quality of life and exert a significant impact on cognitive impairment. Evidence from recent studies suggests that AR may harm children’s neurodevelopment and cognitive function, while both AR and CRS may impact adult cognitive function. According to recent research, the leading cause of mental impairment is thought to be sleep disturbances. Facial symptoms, the nose-brain communication axis, and brain activity and connectivity have also been studied; however, basic research is still lacking and needs to be expanded upon. In terms of treatment, sinus surgery, corticosteroids, and second-generation antihistamines have shown some promise in enhancing cognitive function. However, further research is necessary to comprehend the impact of other drugs on cognition and the underlying mechanisms.

Current basic research remains in its early stages. Future studies may focus on inflammatory signaling pathways such as TLR4/NF-κβ and glial cells (including microglia and astrocytes) to further explore the synergistic effects of inflammatory and neural signals within the nose-brain communication axis. In clinical research, substantial findings have been reported. However, most studies on the relationship between AR, CRS and cognitive impairment are based on cross-sectional or case–control designs. Many of these studies lack adequate control for comorbidities, seasonal effects and pharmacotherapies, and there is no uniformity in the assessment tools for cognitive function. Future research should adopt more rigorous experimental designs to exclude confounding factors and verify the potential causal relationship between AR/CRS and cognitive impairment.

In clinical practice, given the impact of comorbid symptoms associated with AR and CRS on cognitive impairment, symptom management strategies should be implemented. This includes targeted interventions for symptoms such as anxiety, depression, and pain. Additionally, careful selection of medications, dosages, and administration routes can help minimize adverse effects on cognitive function.

## References

[1] Safia A Elhadi UA Karam M Merchavy S Khater A. A meta-analysis of the prevalence and risk of mental health problems in allergic rhinitis patients. J Psychosom Res. (2024) 184:111813. doi: 10.1016/j.jpsychores.2024.111813, 38871533

[2] Pang K Li G Li M Zhang L Fu Q Liu K . Prevalence and risk factors for allergic rhinitis in China: a systematic review and Meta-analysis. Evid Based Complement Alternat Med. (2022) 2022:1–14. doi: 10.1155/2022/7165627 PMC9525776 36193147

[3] Licari A Magri P de A Giannetti A Indolfi C Mori F . Epidemiology of allergic rhinitis in children: a systematic review and Meta-analysis. J Allergy Clin Immunol Pract. (2023) 11:2547–56. doi: 10.1016/j.jaip.2023.05.016 37236349

[4] Savoure M Bousquet J Jaakkola JJK Jaakkola MS Jacquemin B Nadif R . Worldwide prevalence of rhinitis in adults: a review of definitions and temporal evolution. Clin Transl Allergy. (2022) 12:e12130. doi: 10.1002/clt2.12130, 35344304 PMC8967272

[5] Li S Wu W Wang G Zhang X Guo Q Wang B . Association between exposure to air pollution and risk of allergic rhinitis: a systematic review and meta-analysis. Environ Res. (2022) 205:112472. doi: 10.1016/j.envres.2021.112472 34863689

[6] Zhang S Fu Q Wang S Jin X Tan J Ding K . Association between air pollution and the prevalence of allergic rhinitis in Chinese children: a systematic review and meta-analysis. Allergy Asthma Proc. (2022) 43:e47–57. doi: 10.2500/aap.2022.43.220044, 36065105

[7] Gong X Han Z Fan H Wu Y He Y Fu Y . The interplay of inflammation and remodeling in the pathogenesis of chronic rhinosinusitis: current understanding and future directions. Front Immunol. (2023) 14:1238673. doi: 10.3389/fimmu.2023.1238673, 37771597 PMC10523020

[8] Schleimer RP. Immunopathogenesis of chronic rhinosinusitis and nasal polyposis. Annu Rev Pathol. (2017) 12:331–57. doi: 10.1146/annurev-pathol-052016-100401, 27959637 PMC5514544

[9] Corren J. Inflammatory disorders associated with allergy: overview of Immunopathogenesis and implications for treatment. Immunol Allergy Clin N Am. (2017) 37:233–46. doi: 10.1016/j.iac.2017.01.001 28366474

[10] Lam K Schleimer R Kern RC. The etiology and pathogenesis of chronic rhinosinusitis: a review of current hypotheses. Curr Allergy Asthma Rep. (2015) 15:41. doi: 10.1007/s11882-015-0540-2, 26143392 PMC4874491

[11] Kato A Schleimer RP Bleier BS. Mechanisms and pathogenesis of chronic rhinosinusitis. J Allergy Clin Immunol. (2022) 149:1491–503. doi: 10.1016/j.jaci.2022.02.016, 35245537 PMC9081253

[12] Mullol J Azar A Buchheit KM Hopkins C Bernstein JA. Chronic rhinosinusitis with nasal polyps: quality of life in the biologics era. J Allergy Clin Immunol Pract. (2022) 10:1434–1453.e9. doi: 10.1016/j.jaip.2022.03.002, 35306180

[13] Bai J Tan BK. B lineage cells and IgE in allergic rhinitis and CRSwNP and the role of Omalizumab treatment. Am J Rhinol Allergy. (2023) 37:182–92. doi: 10.1177/19458924221147770, 36848269 PMC10830379

[14] Keeley T Gaw N Ahmed W Alfonso-Cristancho R Sousa AR Forde K . Chronic rhinosinusitis with nasal polyps (CRSwNP) symptom verbal response scales: content validity testing for use in adults with CRSwNP. J Patient Rep Outcomes. (2024) 8:152. doi: 10.1186/s41687-024-00827-4, 39704924 PMC11662121

[15] Chee J Pang KW Low T Wang Y Subramaniam S. Epidemiology and aetiology of chronic rhinosinusitis in Asia—a narrative review. Clin Otolaryngol. (2022) 48:305–12. doi: 10.1111/coa.13971, 35997660

[16] Min HK Lee S Kim S Son Y Park J Kim HJ . Global incidence and prevalence of chronic rhinosinusitis: a systematic review. Clin Exp Allergy. (2025) 55:52–66. doi: 10.1111/cea.14592, 39506931

[17] Bhattacharyya N. Contemporary incremental healthcare costs for chronic rhinosinusitis in the United States. Laryngoscope. (2021) 131:2169–72. doi: 10.1002/lary.29454, 33606274

[18] Ma C Mehta NK Nguyen SA Gudis DA Miglani A Schlosser RJ. Demographic variation in chronic rhinosinusitis by subtype and region: a systematic review. Am J Rhinol Allergy. (2022) 36:367–77. doi: 10.1177/19458924211056294, 34825572

[19] Sedaghat AR Kuan EC Scadding GK. Epidemiology of chronic rhinosinusitis: prevalence and risk factors. The journal of allergy and clinical immunology. In Pract. (2022) 10:1395–403. doi: 10.1016/j.jaip.2022.01.016, 35092822

[20] Bhattacharyya N. Functional limitations and workdays lost associated with chronic rhinosinusitis and allergic rhinitis. Am J Rhinol Allergy. (2012) 26:120–2. doi: 10.2500/ajra.2012.26.3752, 22487288 PMC3906503

[21] Settipane RA. Complications of allergic rhinitis. Allergy Asthma Proc. (1999) 20:209–13. doi: 10.2500/108854199778339053 10476318

[22] Marshall PS O'hara C Steinberg P. Effects of seasonal allergic rhinitis on selected cognitive abilities. Ann Allergy Asthma Immunol. (2000) 84:403–10. doi: 10.1016/S1081-1206(10)62273-9 10795648

[23] Jafari A De Lima XL Bernstein JD Simonyan K Bleier BS. Association of Sinonasal Inflammation with Functional Brain Connectivity. JAMA Otolaryngol Head Neck Surg. (2021) 147:534–43. doi: 10.1001/jamaoto.2021.0204, 33830194 PMC8033506

[24] Penchev P Milanova-Ilieva D Gaydarski L Petrov PP Ketev K Stanchev P . Attention deficit and memory function in children with bronchial asthma: a systematic review and Meta-analysis of 104,975 patients with trial sequential analysis. Children (Basel). (2025) 12:1013. doi: 10.3390/children12081013 40868465 PMC12384668

[25] Christopher-Hayes NJ Haynes SC Kenyon NJ Merchant VD Schweitzer JB Ghetti S. Asthma and memory function in children. JAMA Netw Open. (2024) 7:e2442803. doi: 10.1001/jamanetworkopen.2024.42803, 39527060 PMC11555544

[26] Abuaish S Eltayeb H Bepari A Hussain SA Alqahtani RS Alshahrani WS . The Association of Asthma with anxiety, depression, and mild cognitive impairment among middle-aged and elderly individuals in Saudi Arabia. Behav Sci. (2023) 13:842. doi: 10.3390/bs13100842, 37887495 PMC10604786

[27] Ozdoganoglu T Songu M Inancli HM. Quality of life in allergic rhinitis. Ther Adv Respir Dis. (2011) 6:25–39. doi: 10.1177/1753465811424425 22032987

[28] Borres MP. Allergic rhinitis: more than just a stuffy nose. Acta Paediatr. (2009) 98:1088–92. doi: 10.1111/j.1651-2227.2009.01304.x, 19397546

[29] Brawley A Silverman B Kearney S Guanzon D Owens M Bennett H . Allergic rhinitis in children with attention-deficit/hyperactivity disorder. Ann Allergy Asthma Immunol. (2004) 92:663–7. doi: 10.1016/S1081-1206(10)61434-2 15237769

[30] Wei J Li Y Wu Q Lei B Gui X. Bidirectional association between allergic rhinitis and attention-deficit/hyperactivity disorder: a systematic review and meta-analysis. J Affect Disord. (2025) 369:499–507. doi: 10.1016/j.jad.2024.10.032, 39389122

[31] Kremer B Den Hartog HM Jolles J. Relationship between allergic rhinitis, disturbed cognitive functions and psychological well-being. Clin Exp Allergy. (2002) 32:1310–5. doi: 10.1046/j.1365-2745.2002.01483.x, 12220469

[32] Sansone RA Sansone LA. Allergic rhinitis: relationships with anxiety and mood syndromes. Innov Clin Neurosci. (2011) 8:12–7. 21860841 PMC3159540

[33] Kuo HC Chang LS Tsai ZY Wang LJ. Allergic diseases do not impair the cognitive development of children but do damage the mental health of their caregivers. Sci Rep. (2020) 10:13854. doi: 10.1038/s41598-020-70825-1, 32807818 PMC7431564

[34] Yamasaki A Burks CA Bhattacharyya N. Cognitive and quality of life-related burdens of illness in pediatric allergic airway disease. Otolaryngol Head Neck Surg. (2020) 162:566–71. doi: 10.1177/0194599820908202, 32122241

[35] Papapostolou G Kiotseridis H Romberg K Dahl Å Bjermer L Lindgren M . Cognitive dysfunction and quality of life during pollen season in children with seasonal allergic rhinitis. Pediatr Allergy Immunol. (2020) 32:67–76. doi: 10.1111/pai.13328 32767782 PMC7818136

[36] Jackson-Cowan L Cole EF Silverberg JI Lawley LP. Childhood atopic dermatitis is associated with cognitive dysfunction: a National Health Interview Survey study from 2008 to 2018. Ann Allergy Asthma Immunol. (2021) 126:661–5. doi: 10.1016/j.anai.2020.11.008, 33189871

[37] Kim JH Yi YY Ha EK Cha HR Han MY Baek H-S. Neurodevelopment at 6 years of age in children with atopic dermatitis. Allergol Int. (2023) 72:116–27. doi: 10.1016/j.alit.2022.08.002 36058807

[38] Marcotte DE. Allergy test: seasonal allergens and performance in school. J Health Econ. (2015) 40:132–40. doi: 10.1016/j.jhealeco.2015.01.002, 25680109

[39] Bensnes SS. You sneeze, you lose: the impact of pollen exposure on cognitive performance during high-stakes high school exams. J Health Econ. (2016) 49:1–13. doi: 10.1016/j.jhealeco.2016.05.005, 27315202

[40] Robles-Figueroa M Bedolla-Barajas M Morales-Romero J Pulido-Guillén NA Bustos-Gutiérrez LRM. Allergic rhinitis is associated with loss of energy and concentration difficulty: a cross-sectional study. Am J Rhinol Allergy. (2019) 34:108–14. doi: 10.1177/1945892419877554, 31558036

[41] Wilken JA Berkowitz R Kane R. Decrements in vigilance and cognitive functioning associated with ragweed-induced allergic rhinitis. Ann Allergy Asthma Immunol. (2002) 89:372–80. doi: 10.1016/S1081-1206(10)62038-8, 12392381

[42] Meltzer EO Nathan R Derebery J Stang PE Campbell UB Yeh WS . Sleep, quality of life, and productivity impact of nasal symptoms in the United States: findings from the burden of rhinitis in America survey. Allergy Asthma Proc. (2009) 30:244–54. doi: 10.2500/aap.2009.30.3230, 19549425

[43] Cingi CC Sakallıoğlu Ö Muluk NB Cingi C. Does allergic rhinitis affect communication skills in young adults? Eur Arch Otorrinolaringol. (2015) 273:115–21. doi: 10.1007/s00405-015-3531-y, 25647472

[44] Danagoulian S Deza M. Driving under the influence of allergies: the effect of seasonal pollen on traffic fatalities. J Health Econ. (2025) 99:102945. doi: 10.1016/j.jhealeco.2024.102945, 39657375

[45] Hartgerink-Lutgens I Vermeeren A Vuurman E Kremer B. Disturbed cognitive functions after nasal provocation in patients with seasonal allergic rhinitis. Clin Exp Allergy. (2009) 39:500–8. doi: 10.1111/j.1365-2222.2009.03200.x, 19226277

[46] Trikojat K Buske-Kirschbaum A Schmitt J Plessow F. Altered performance in attention tasks in patients with seasonal allergic rhinitis: seasonal dependency and association with disease characteristics. Psychol Med. (2015) 45:1289–99. doi: 10.1017/S0033291714002384, 25273694

[47] Trikojat K Buske-Kirschbaum A Plessow F Schmitt J Fischer R. Memory and multitasking performance during acute allergic inflammation in seasonal allergic rhinitis. Clin Exp Allergy. (2017) 47:479–87. doi: 10.1111/cea.12893 28122395

[48] Corpening B Burgler A Tamasi B . Associations between ambient pollen exposure and measures of cognitive performance. Environ Epidemiol. (2025) 9:e374. doi: 10.1097/EE9.0000000000000374, 40012845 PMC11864305

[49] Den Hartog HM Derix MM Van Bemmel AL Kremer B Jolles J. Cognitive functioning in young and middle-aged unmedicated out-patients with major depression: testing the effort and cognitive speed hypotheses. Psychol Med. (2003) 33:1443–51. doi: 10.1017/S003329170300833X, 14672253

[50] Eriksson UK Gatz M Dickman PW Fratiglioni L Pedersen NL. Asthma, eczema, rhinitis and the risk for dementia. Dement Geriatr Cogn Disord. (2008) 25:148–56. doi: 10.1159/000112729, 18097143

[51] Fereidouni M Rezapour H Saharkhiz M Mahmoudzadeh S Ayadilord M Askari M . A study of the association of cognitive abilities and emotional function with allergic disorders in young women. BMC Womens Health. (2021) 21:205. doi: 10.1186/s12905-021-01345-x, 34001075 PMC8130253

[52] Soler ZM Eckert MA Storck K Schlosser RJ. Cognitive function in chronic rhinosinusitis: a controlled clinical study. Int Forum Allergy Rhinol. (2015) 5:1010–7. doi: 10.1002/alr.21581 26121963

[53] Cvancara DJ Wood HA Aboueisha M Marshall TB Kao TC Phillips JO . Cognition and saccadic eye movement performance are impaired in chronic rhinosinusitis. Int Forum Allergy Rhinol. (2024) 14:1206–17. doi: 10.1002/alr.23320, 38268115 PMC11789658

[54] Tarasidis GS Deconde AS Mace JC Ashby S Smith TL Orlandi RR . Cognitive dysfunction associated with pain and quality of life in chronic rhinosinusitis. Int Forum Allergy Rhinol. (2015) 5:1004–9. doi: 10.1002/alr.21578, 26246436 PMC4688255

[55] Chang F Hong J Yuan F Wu D. Association between cognition and olfaction-specific parameters in patients with chronic rhinosinusitis. Eur Arch Otorrinolaringol. (2023) 280:3249–58. doi: 10.1007/s00405-023-07853-w, 36689021

[56] Schlosser RJ Storck K Cortese BM Uhde TW Rudmik L Soler ZM. Depression in chronic rhinosinusitis: a controlled cohort study. Am J Rhinol Allergy. (2016) 30:128–33. doi: 10.2500/ajra.2016.30.4290, 26980393 PMC5554332

[57] Mahdavinia M Schleimer RP Keshavarzian A. Sleep disruption in chronic rhinosinusitis. Expert Rev Anti-Infect Ther. (2017) 15:457–65. doi: 10.1080/14787210.2017.1294063 28276943 PMC5967413

[58] Fried J Yuen E Li A Zhang K Nguyen SA Gudis DA . Rhinologic disease and its impact on sleep: a systematic review. Int Forum Allergy Rhinol. (2021) 11:1074–86. doi: 10.1002/alr.22740 33275331

[59] Pratt EL Craig TJ. Assessing outcomes from the sleep disturbance associated with rhinitis. Curr Opin Allergy Clin Immunol. (2007) 7:249–56. doi: 10.1097/ACI.0b013e3280f3c09f 17489043

[60] Yang T Wang HR Mou YK Liu WC Wang Y Song XY . Mutual influence between allergic rhinitis and sleep: factors, mechanisms, and interventions-a narrative review. Nat Sci Sleep. (2024) 16:1451–67. doi: 10.2147/NSS.S482258, 39318396 PMC11420902

[61] Christopher-Hayes NJ Ghetti S. Neurocognitive risks of asthma during childhood. Dev Cogn Neurosci. (2025) 73:101564. doi: 10.1016/j.dcn.2025.101564, 40349572 PMC12139513

[62] Wang Y Song XY Wei SZ Wang HR Zhang WB Li YM . Brain response in allergic rhinitis: profile and proposal. J Neurosci Res. (2023) 101:480–91. doi: 10.1002/jnr.25159, 36564932

[63] Parsazadegan T Salimi M Ghazvineh S Raoufy MR. Cognitive disorders in allergic rhinitis may be induced by decline of respiration entrained rhythm in the brain. Med Hypotheses. (2018) 121:89–90. doi: 10.1016/j.mehy.2018.09.037, 30396502

[64] Salimi M Ghazvineh S Zare M Parsazadegan T Dehdar K Nazari M . Distraction of olfactory bulb-medial prefrontal cortex circuit may induce anxiety-like behavior in allergic rhinitis. PLoS One. (2019) 14:e0221978. doi: 10.1371/journal.pone.0221978, 31509547 PMC6738655

[65] Rodrigues J Rocha MI Teixeira F Resende B Cardoso A Sá SI . Structural, functional and behavioral impact of allergic rhinitis on olfactory pathway and prefrontal cortex. Physiol Behav. (2023) 265:114171. doi: 10.1016/j.physbeh.2023.114171, 36965572

[66] Ebrahim Soltani Z Badripour A Haddadi NS Elahi M Kazemi K Afshari . Allergic rhinitis in BALB/c mice is associated with behavioral and hippocampus changes and neuroinflammation via the TLR4/ NF-kappaB signaling pathway. Int Immunopharmacol. (2022) 108:108725. doi: 10.1016/j.intimp.2022.108725, 35381564

[67] Harrass S Yi C Chen H. Chronic rhinosinusitis and Alzheimer’s disease—a possible role for the nasal microbiome in causing neurodegeneration in the elderly. Int J Mol Sci. (2021) 22:11207. doi: 10.3390/ijms222011207, 34681867 PMC8541405

[68] Gao Z Chen X Xiang R Zhang W Tan L Fan W . Changes in resting-state spontaneous brain activity in patients with allergic rhinitis: a pilot neuroimaging study. Front Neurosci. (2021) 15:697299. doi: 10.3389/fnins.2021.697299, 34335172 PMC8317644

[69] Wang T Huang X Wang J. Asthma's effect on brain connectivity and cognitive decline. Front Neurol. (2023) 13:1065942. doi: 10.3389/fneur.2022.1065942, 36818725 PMC9936195

[70] Jauregui I Mullol J Davila I Ferrer M Bartra J del Cuvillo A . Allergic rhinitis and school performance. J Investig Allergol Clin Immunol. (2009) 19:32–9. 19476052

[71] Alt JA Mace JC Smith TL Soler ZM. Endoscopic sinus surgery improves cognitive dysfunction in patients with chronic rhinosinusitis. Int Forum Allergy Rhinol. (2016) 6:1264–72. doi: 10.1002/alr.21820, 27384037 PMC5140732

[72] Kay GG. The effects of antihistamines on cognition and performance. J Allergy Clin Immunol. (2000) 105:S622–7. doi: 10.1067/mai.2000.106153 10856168

[73] Gandon JM Allain H. Lack of effect of single and repeated doses of levocetirizine, a new antihistamine drug, on cognitive and psychomotor functions in healthy volunteers. Br J Clin Pharmacol. (2002) 54:51–8. doi: 10.1046/j.1365-2125.2002.01611.x, 12100225 PMC1874390

[74] Wilken JA Kane RL Ellis AK Rafeiro E Briscoe MP Sullivan CL . A comparison of the effect of diphenhydramine and desloratadine on vigilance and cognitive function during treatment of ragweed-induced allergic rhinitis. Ann Allergy Asthma Immunol. (2003) 91:375–85. doi: 10.1016/S1081-1206(10)61685-7, 14582817

[75] Valk PJL Simons R Jetten AM Valiente R Labeaga L. Cognitive performance effects of Bilastine 20 mg during 6 hours at 8000 ft cabin altitude. Aerospace Med Human Perform. (2016) 87:622–7. doi: 10.3357/AMHP.4522.2016, 27503042

[76] Blaiss MS. Allergic rhinitis and impairment issues in school children: a consensus report. Curr Med Res Opin. (2004) 20:1937–52. doi: 10.1185/030079904X13266, 15704310

[77] Reményi Á Grósz A Szabó SA Tótka Z Molnár D Helfferich F. Comparative study of the effect of bilastine and cetirizine on cognitive functions at ground level and at an altitude of 4,000 m simulated in hypobaric chamber: a randomized, double-blind, placebo-controlled, cross-over study. Expert Opin Drug Saf. (2018) 17:859–68. doi: 10.1080/14740338.2018.1502268, 30032673

[78] Vacchiano C Moore J Rice GM Crawley G. Fexofenadine effects on cognitive performance in aviators at ground level and simulated altitude. Aviat Space Environ Med. (2008) 79:754–60. doi: 10.3357/ASEM.2212.2008, 18717113

[79] Philpot EE. Safety of second generation antihistamines. Allergy Asthma Proc. (2000) 21:15–20. doi: 10.2500/108854100778249033 10748947

[80] Spangler DL Brunton S. Efficacy and central nervous system impairment of newer-generation prescription antihistamines in seasonal allergic rhinitis. South Med J. (2006) 99:593–9. doi: 10.1097/01.smj.0000221631.98056.87, 16800414

[81] Mansfield LE Posey CR. Daytime sleepiness and cognitive performance improve in seasonal allergic rhinitis treated with intranasal fluticasone propionate. Allergy Asthma Proc. (2007) 28:226–9. doi: 10.2500/aap.2007.28.2950, 17479609

[82] Sharma S Karki D Julitta K. Effect of long-term inhaled corticosteroids therapy on cognitive function in patients with bronchial asthma and chronic obstructive pulmonary disease. Lung India. (2024) 41:357–61. doi: 10.4103/lungindia.lungindia_399_23, 39215978 PMC11472989

[83] Rowan NR Schlosser RJ Storck KA Ganjaei KG Soler ZM. The impact of medical therapy on cognitive dysfunction in chronic rhinosinusitis. Int Forum Allergy Rhinol. (2019) 9:738–45. doi: 10.1002/alr.22323, 30811873

[84] Yoo F Schlosser RJ Storck KA Ganjaei KG Rowan NR Soler ZM. Effects of endoscopic sinus surgery on objective and subjective measures of cognitive dysfunction in chronic rhinosinusitis. Int Forum Allergy Rhinol. (2019) 9:1135–43. doi: 10.1002/alr.22406, 31449738

